# Rapid Screening of Gene Function by Systemic Delivery of Morpholino Oligonucleotides to Live Mouse Embryos

**DOI:** 10.1371/journal.pone.0114932

**Published:** 2015-01-28

**Authors:** Kathryn S. McClelland, Elanor N. Wainwright, Josephine Bowles, Peter Koopman

**Affiliations:** Institute for Molecular Bioscience, The University of Queensland, Brisbane, Queensland, Australia; Laboratoire de Biologie du Développement de Villefranche-sur-Mer, FRANCE

## Abstract

Traditional gene targeting methods in mice are complex and time consuming, especially when conditional deletion methods are required. Here, we describe a novel technique for assessing gene function by injection of modified antisense morpholino oligonucleotides (MOs) into the heart of mid-gestation mouse embryos. After allowing MOs to circulate through the embryonic vasculature, target tissues were explanted, cultured and analysed for expression of key markers. We established proof-of-principle by partially phenocopying known gene knockout phenotypes in the fetal gonads (*Stra8*, *Sox9*) and pancreas (*Sox9*). We also generated a novel double knockdown of *Gli1* and *Gli2*, revealing defects in Leydig cell differentiation in the fetal testis. Finally, we gained insight into the roles of *Adamts19* and *Ctrb1*, genes of unknown function in sex determination and gonadal development. These studies reveal the utility of this method as a means of first-pass analysis of gene function during organogenesis before committing to detailed genetic analysis.

## Introduction

One of the central challenges in the era of functional genomics is to understand gene function and unravel the complex networks in which proteins operate to determine phenotype. With RNA-seq data amassing on top of an already large list of genes gleaned from microarray screens, many candidate genes now require functional assessment. In addition, possible causative genes for human developmental diseases are being identified rapidly in rare disease cohorts as a result of whole exome and whole genome sequencing.

Much of the functional genomics effort focuses on the mouse model because of its relevance to human development, physiology and disease. Investigation of gene function in mouse has traditionally involved the generation and breeding of complete or conditional loss-of-function alleles via homologous recombination, involving a complex and time-consuming experimental pipeline. Even with advances in genome editing technologies such as the CRISPR/Cas-9 system (for review see [1]), the generation of knockout animals for every promising gene candidate is impractical. Moreover, it is often the case that, after investing the time and resources required to generate a conventional or conditional gene knockout, little or no phenotype results. Therefore, there is a pressing need to develop methods that provide insight into developmental gene function either as a pre-screen before committing to genome manipulation approaches *in vivo*, or as a means of prioritizing candidates for further analysis.

With this goal in mind, a variety of methods for accelerated *ex vivo* functional analysis have been reported. In the field of gonadal development, these methods have included injection, electroporation or liposome-based delivery of viral-based or siRNA-based constructs into explanted tissue [2–4], followed by organ culture and histological or molecular analysis. Typically, these approaches have caused damage to the target tissue as well as being limited in delivery area. For other developing organs, such as mouse lung and kidney, morpholino antisense oligonucleotides (MOs) have been added to the culture media, but these experiments show high variability due to limited passive uptake of the MO [5–9].

We aimed to develop a method whereby gene function could be perturbed *ex vivo*, rapidly and without injury to the target organ. Here we show that injection of commercially available MOs into the beating heart of a 11.5 dpc (days *post coitum*) mouse embryo results in delivery via the vasculature to the gonads and pancreas. We demonstrate knockdown of protein expression for a number of target genes, leading to predicted downstream effects for known genes and novel functional insights for other genes or combinations of genes. This method offers a rapid, reproducible, efficient means of rapidly pre-screening gene candidates for likely function, as a prelude to more rigorous functional studies in mice.

## Materials and Methods

### Morpholino design

**Fig 1:**
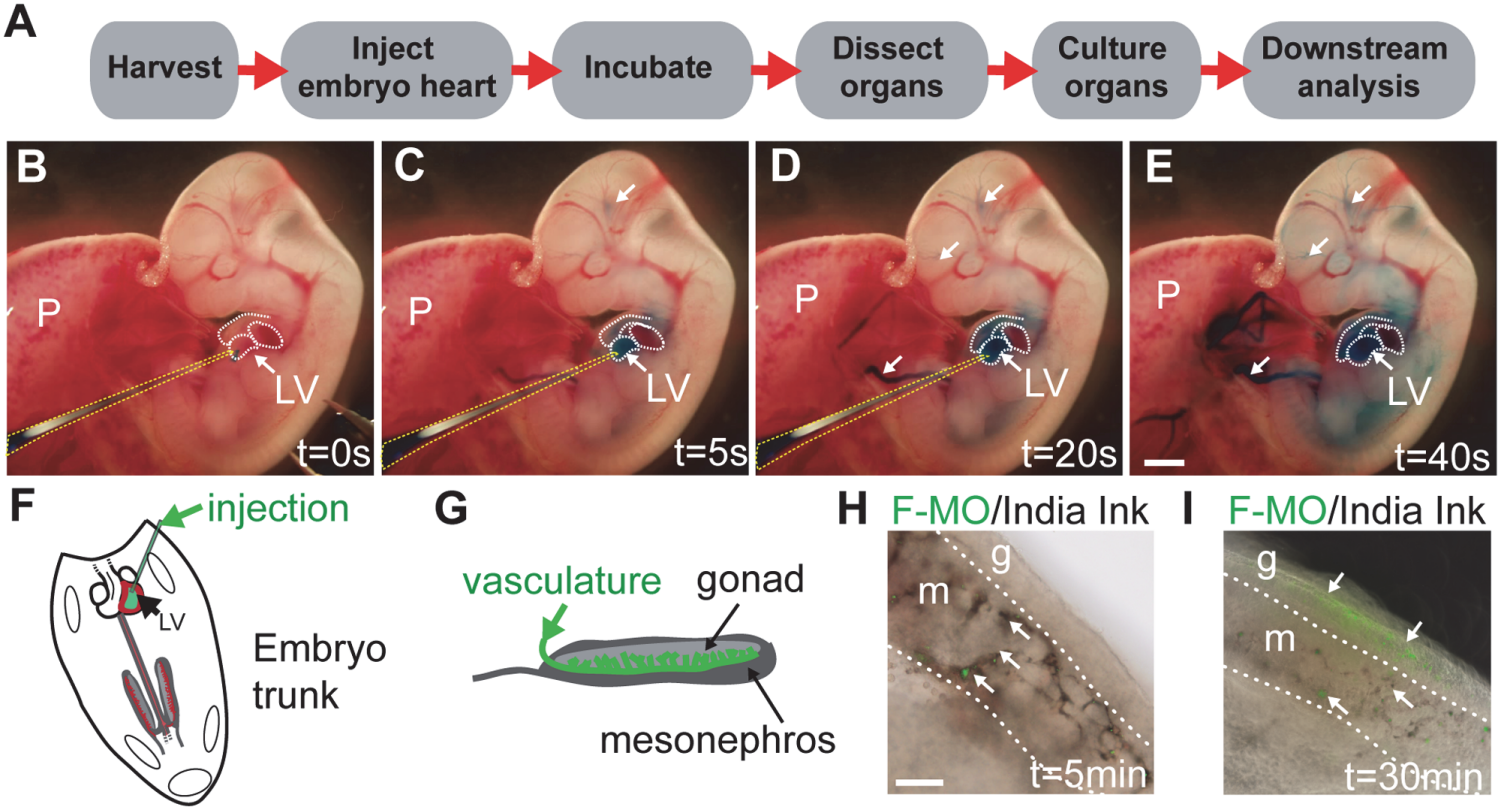
Overview of method: MO delivery by heart injection. (A) Experimental pipeline from harvest of embryos through to injection, culture and downstream analyses. Visualisation of heart injection protocol can be seen in S1 Video and images B–E. The cocktail of dye and MO in PBS is delivered via injection into the left ventricle of the beating heart at 11.5 dpc (B). Dye can be visualised going around the embryonic vasculature (indicated by white arrows) (C, D) and into the head vasculature (D) before the whole embryo is coloured (E). Schematic of ventricle injection (F) and the embryonic gonad which is highly vascularised (G). Delivery of India ink and F-MO (indicated by white arrows) shows the compounds reaching the mesonephric plexus at 5 min post-injection (H; *n* = 3); after 30 min F-MO positive cells were observed in the gonad proper (I; *n* = 3). s = seconds; min = minutes; g = gonad; m = mesonephros; F-MO = carboxyfluorescein-labelled standard control morpholino oligonucleotide. Scale bars: E = 1 mm, H = 0.5 mm.

Splice site MOs were designed to target exon/intron boundaries of target genes (for sequences see S1 Table). All MOs were vivo-MOs which incorporate a dendrimeric octaguanidine delivery moiety end modification, with the exception shown in Fig. 1H, I, where a carboxyfluorescein-labelled standard control MO (F-MO) was used.

### Heart injections

For ease of sexing embryos, we used the X-linked GFP line (Hadjantonakis et al., 1998), maintained on an outbred Swiss albino background (Quackenbush strain). Noon on the day on which the mating plug was detected was designated as 0.5 days *post coitum* (dpc). All animal work was conducted according to protocols approved by the University of Queensland Animal Ethics Committee. This study was approved by the University of Queensland Animal Ethics Committee (Permit Number: IMB/176/13/NHMRC/ARC).

Embryos were explanted at 11.5 dpc and placed into PBS (phosphate buffered saline) at 37°C with the amniotic sac intact and the placenta attached. If required, embryos were sexed by GFP expression. The amniotic sac was opened, taking care not to damage any major blood vessels. The left ventricle of the beating heart of the embryo was injected with a MO-cocktail (20 ng/μL (single target) or 15 ng/μL (per MO, two targets) and 6% commercial food dye (Queen Fine Foods Pty. Ltd.)). For each embryo either control or MO targeted against gene of interest (Gene Tools, LLC) was delivered using a Sutter-pulled glass capillary needle. Injection was continued until the marker dye was observed in the head vein (approx. 6–8 heart beats, equivalent to ~20–27 ng MO/embryo (single MO) or ~30–40 ng MO/embryo (combination of two MOs); see S1 Video and Fig. 1). Embryos with non-beating or weakly beating hearts, or where injection was unsuccessful as judged by lack of circulation of the dye (about 1 in 15 embryos), were excluded from further study. Embryos were left to recover for 30 min in pre-warmed PBS in an incubator at 37°C, 5% CO_2_; hearts were still beating at the end of this period. Video of the above procedure was captured on an Olympus SZX-12 Stereomicroscope (see S1 Video).

For assessing delivery area using a F-MO: 20 μg/mL F-MO (20–27 ng/embryo) and 2% India ink was delivered by heart injection as described above (*n* = 3). After 5 and 30 min recovery the genital ridge was imaged on an Olympus BX-51 Upright Fluorescence/Brightfield microscope.

For gonad culture, UGR (urogenital ridge: gonad plus mesonephros) was dissected out and hanging drops were prepared by pipetting 40 μL of media (BJGB media (Gibco) with 4% Serum Supreme (Lonza), 1% penicillin/streptomycin (Gibco) and 200 mg/mL ascorbic acid (Sigma Aldrich)) containing a single UGR onto the inner face of the lid of a 24 well tissue culture plate. PBS (500 μL) was added to each well and the lid was then inverted to close the plate. After 48 h, cultured gonads were washed in PBS for 5 min and processed for qRT-PCR, Western blot or immunohistochemistry.

For pancreas culture, the foregut endoderm was isolated and any non-affiliated organs removed. The foregut was placed on a Millipore (5 μM TPMT) filter floating on 600 μl of culture medium (M199 media (Gibco) with 10% Serum Supreme (Lonza) and 2% penicillin/streptomycin (Gibco)) and cultured for 4–6 days at 37°C, 5% CO_2_ with the media changed every other day. After culture, tissues were washed in PBS for 5 min and processed for qRT-PCR or immunohistochemistry.

### Quantitative RT-PCR analysis

Total RNA was extracted and cDNA generated from cultured gonad or pancreas as previously described (Bowles et al., 2010). Duplicate assays were carried out on an ABI Prism 7500 Sequence Detector System. *Tbp* (TATA box binding protein) was used as an endogenous control to normalize gene expression levels [10]. Taqman gene expression sets were as listed in S2 Table.

Relative transcript abundance was calculated using the 2^−ΔCT^ method. Error bars represent S.E.M. calculated from independent biological replicates; statistical significance was assessed using unpaired (two-tailed) Student’s *t*-test.

### Immunofluorescence

Analyses were carried out on fixed, paraffin-embedded 7 μm sections using standard methods. Briefly, gonad plus mesonephros complexes or foreguts were fixed in 4% paraformaldehyde in phosphate buffered saline overnight at 4°C. Tissues were embedded in 1.5% low melt agarose, ethanol dehydrated, paraffin-embedded and 7 μm sections were cut using a Leica Microtome. Slides were dewaxed by 2 x 10 min washes in xylene, re‐hydrated and boiled for 5 min in Antigen Unmasking Solution (Vector Laboratories), then incubated at room temperature for 60 min. The slides were washed for 3 x 10 min in 0.1% Triton-X in PBS (PBTX). The sections were incubated with primary antibodies, which were diluted in blocking buffer at 4°C overnight (for primary antibodies see S3 Table). Antibodies were removed with three washes in PBTX, and the slides re‐blocked for 30 min at room temperature. Secondary antibodies were incubated at room temperature for 2 h. The secondary antibodies were removed with three PBS washes before DAPI staining and mounting with a 60% glycerol/PBS solution. Secondary antibodies were all from Invitrogen Molecular Probes (see S4 Table). Sections were examined by confocal microscopy using a Zeiss LSM-510 META or LSM-710 META confocal microscope.

### Whole-mount immunofluorescence

Whole mount immunofluorescence was performed as detailed in [11].

### Cell quantification

For quantification of the number of INS- and PAX6-positive cells in the pancreas, and HSD3β, NR5A1, SOX9-positive cells in the XY gonad, de-identified gonads or foreguts were serially sectioned at 7 μm and processed as per the immunofluorescence protocol. Quantification was performed on all sections per sample using the ImageJ64 “Cell Counter” plugin. Error bars depict S.E.M. calculated from independent biological replicates; statistical significance was determined using unpaired (two-tailed) Student’s *t*-test. Asterisks indicate level of statistical significance in pertinent comparisons.

### Western blot

Western blots were carried out as described previously [12], with slight modifications. Briefly, gonad pairs were dissociated with a 13-gauge needle and lysed in 1× SDS sample buffer (62.5 mM Tris—HCl (pH 6.8), 2% SDS, 10% glycerol, 50 mM dithiothreitol, and 0.01% w/v bromophenol blue), separated on SDS-PAGE and transferred to a PVDF membrane (Millipore). SOX9 primary antibody was incubated for 2 h at room temperature and then overnight at 4°C with 13.5 dpc testis as a positive control and 13.5 dpc ovary as a negative control. For primary antibodies see S3 Table, for secondary antibodies see S4 Table. Proteins were visualized using Clarity Western ECL Substrate (Bio-Rad) on a ChemiDoc machine (Bio-Rad). Raw intensity of bands was determined using Image Lab Software (version 4.0). SOX9 intensity units were calculated relative to α-TUB or β-ACT loading control and relative downregulation calculated with cMO sample set to 1 for individual cMO vs. Sox9MO-treated samples on each of 3 blots. Error bars represent S.E.M. calculated from independent biological replicates; statistical significance was assessed using unpaired (two-tailed) Student’s *t*-test.

### Flow cytometry and cell sorting

Flow cytometry and cell sorting was carried out as described previously [13]. Briefly, 12.5 dpc *Sf1*-eGFP [14] litters were dissected, gonads sexed by eye and separated from the mesonephros before being dissociated. Cells were incubated with SSEA1-PE antibody (BD Biosciences) to tag germ cells. FACS was performed using a BD FACS Aria cell sorter. Pools of germ (SSEA1-positive) and eGFP-positive cells were collected separately and total RNA was extracted and cDNA prepared as described [15]. Cells from three or four independent litters and sorting experiments were used for qRT-PCR analysis.

## Results

### Method development: Delivering morpholinos to fetal organs

Initially, we trialled the inclusion of standard ‘naked’ MOs or vivo-MOs (in which the MO is linked to a dendrimeric octaguanidine delivery moiety) in the media for *ex vivo* organ culture from 11.5 dpc for 48h (data not shown), using a protocol similar to those previously published for lungs and kidneys [7,9], but were unable to achieve widespread tissue uptake and hence efficacy. Therefore, we developed a novel protocol that relied on a combination of two approaches.

First, in order to deliver the compounds uniformly through the organs of interest in the mid-gestation embryo, we looked to classic experiments in mouse and chick, where India ink was used to visualise the early vasculature (for review see [16]). This approach has also been utilised to deliver siRNA and viral constructs to the embryo [17], and to deliver lectin to the 11.5 dpc gonad via the vasculature [18]. These studies relied on injection of compounds into the beating embryonic heart, and so we reasoned that this approach might offer a way to successfully deliver MOs to vascularised tissues in the mouse embryo.

Second, Vivo-Morpholinos (Gene Tools, LLC) were chosen for injection as they reportedly show improved systemic delivery efficacy compared to standard MOs [19–21]. Oligonucleotides were designed to span intron/exon boundaries within the pre-mRNA to produce non-functional, mis-spliced gene products. A standard commercial 25-mer MO (see Materials and Methods) was used as a control for the specificity of MO effects.

We trialled our knockdown procedure using the developing ovaries, testes and pancreas as a test-bed. These organs are well suited to vascular delivery of compounds, are readily explanted, develop normally in organ culture, and are well characterised in terms of morphological and molecular markers of differentiation and morphogenesis. Examination of organogenesis allows specificity and off-target effects of the MO to be assessed by testing for markers of differentiation of the targeted cell type and multiple non-targeted other cell types. Inclusion of developing gonads in these studies offers the additional advantage that known differences in sexually dimorphic gene expression can be used as a further control for general toxicity and/or off-target effects.

A summary of the workflow is shown in Fig. 1A, and the detailed protocol is described in Materials and Methods. Conceptuses were explanted at 11.5 dpc and the amniotic sac of individual embryos opened, taking care not to disrupt major amniotic blood vessels. A MO/food dye cocktail was injected into the left ventricle of the beating heart (Fig. 1B, F) until the dye was observed to travel around the embryo and into the vessels in the head, typically after 6–10 heart beats (Fig. 1C–E; S1 Video). After injection, embryos were allowed to recover for ~30 min to enable delivery of MO throughout the vasculature. Subsequently, tissues of interest were explanted, and cultured *ex vivo*, before detailed analysis of gene and protein marker expression. In preliminary experiments, we used carboxyfluorescein-labelled MO (F-MO) to assess the extent of delivery to tissues (*n* = 3). In the case of the developing gonads, F-MO and India ink were observed in the nascent mesonephric vasculature at 5 min post-injection (Fig. 1G, H) and were clearly visible in the gonadal tissue after 30 min (Fig. 1I), suggesting that the dye and MO had accessed the target tissue.

### Proof of principle: STRA8 in the developing ovary

The germ cells of the gonad are the precursors of sperm (XY) or oocytes (XX): whether they adopt the male or female developmental pathway is determined by their somatic environment (for review see [22]). Upregulation of the gatekeeping gene *Stra8* (*stimulated by retinoic acid gene 8*) at 12.5 dpc is essential for germ cell entry into meiosis in the developing ovary, as demonstrated by the blockade of meiosis in XX *Stra8* ^-/-^ gonads (Baltus et al., 2006; for review see [23]). Since *Stra8* ^-/-^ gonads have a well-defined phenotype, we tested MO knockdown of *Stra8* as a proof-of-principle experiment.

**Fig 2:**
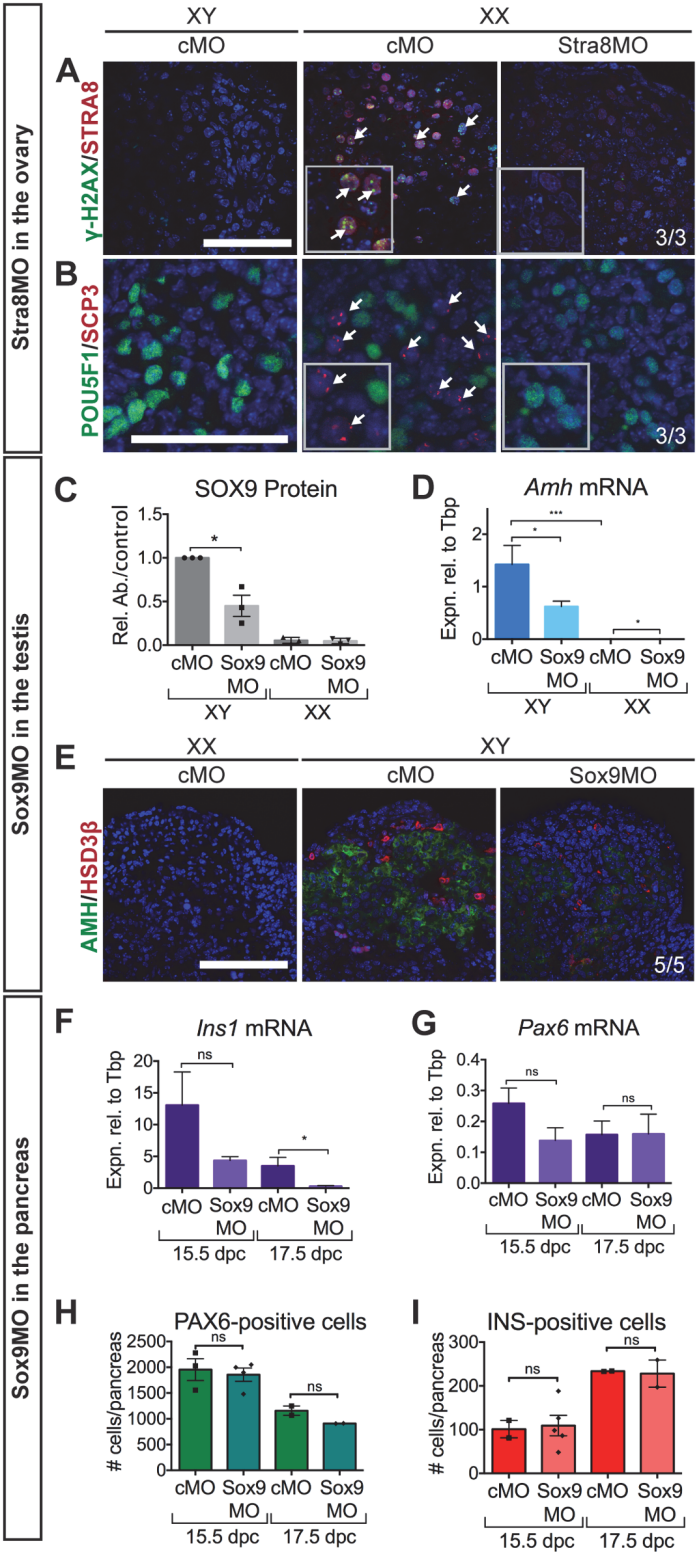
Partial phenocopy of known gene knockouts in gonad and pancreas. (A, B) STRA8 knockdown: IF showed knockdown of STRA8 (A) in Stra8MO-treated XX gonads. Nuclear localisation of meiosis markers (γH2AX (A) and SCP3 (B); indicated by white arrows; see inserts) was absent but germ cells were present (POU5F1 (B); see inserts) in XX Stra8MO-treated gonads. (C–E) Knockdown of SOX9 in the gonad: Western blot for SOX9 (relative to α-TUBULIN or β-ACTIN) showed a downregulation of SOX9 (C) after Sox9MO treatment in XY gonads (*n* = 3). Downregulation of expression of SOX9 target gene *Amh* (D) expression was observed by qRT-PCR (*n* = 8, 15, 11, 4). IF for AMH and HSD3β (E) showed that AMH staining was weaker in XY Sox9MO samples compared to XY controls and that HSD3β-positive FLCs were present but staining was weaker in XY Sox9MO-treated gonads. (F–I) Knockdown of SOX9 in the pancreas: qRT-PCR of Sox9Mo treated pancreata showed *Ins1* (F) was downregulated but *Pax6* (G) was unchanged (*n* = 5, 5, 5, 5). Quantification of PAX6/INS-positive cells revealed that PAX6-positive (H) and INS-positive (I) cell number was unaltered by Sox9MO treatment (*n* = 3, 4, 2, 2). Scale bars = 100 μM; cMO = control morpholino; xMO = morpholino targeting gene x. For Western blots SOX9 levels were normalised to α-TUBULIN or β-ACTIN loading controls and Sox9MO-treated XY gonads measured relative to cMO treated XY gonads with expression for each blot set to 1. Rel. Ab./control = Relative Abundance of SOX9 to α-TUBULIN or β-ACTIN. For all qRT-PCR levels are shown relative to *Tbp*, error = S.E.M. For cell quantification error = S.E.M. with individual counts plotted. * = p = 0.05, ** = p = 0.001, *** = p = 0.0001, ns = not statistically significant.

Although *Stra8* transcript could still be detected after MO treatment (Part A in S1 Fig.), expression of STRA8 protein was greatly decreased as measured by immunofluorescence in Stra8MO-treated XX gonads, indicating successful knockdown (Fig. 2A). Strikingly, meiotic markers γ-H2AX (H2A histone family, member X; Fig. 2A) and SCP3 (synaptonemal complex protein 3; Fig. 2B) were not localised to the nucleus in XX Stra8MO samples, in contrast to control ovaries, where germ cells began to show these hallmark signs of entry into meiosis. Stra8MO knockdown did not have a direct effect on qRT-PCR expression of other meiosis markers (Part C–E in S1 Fig.). However, functional aspects of meiosis, such as SCP3 nuclear localisation, were clearly affected by Stra8MO treatment (Fig. 2 A, B).

We tested for possible effects of generalised toxicity in MO-treated gonads by examining expression of a range of cell lineage markers. Immunofluorescence and qRT-PCR for markers of germ cells—OCT4/POU5F1 (POU domain, class 5, transcription factor 1; Fig. 2B, Part G in S1 Fig.), *Mvh/Ddx4* (Deadbox polypeptide 4; Part F in S1 Fig.) and CDH1 (Cadherin 1; Part H, I in S1 Fig.) indicated that the number of germ cells was unaffected in Stra8MO treated gonads, suggesting no qualitative or quantitative detrimental effect on germ cells. Furthermore, expression of the somatic marker FOXL2 (Forkhead box L2; Part B, I in S1 Fig.) was unchanged in Stra8MO XX gonads, indicating that gonadogenesis in general was not impaired by MO-treatment. Combined, these data show that the reduced meiotic marker expression was likely a specific consequence of MO antagonism of STRA8 expression, rather than generalised toxicity.

In summary, the suppression of markers associated with meiotic entry suggests that germ cells failed to successfully enter meiosis in Stra8MO knockdown XX gonads. Thus, the Stra8MO knockdown partially phenocopied the *Stra8* ^-/-^ gonad phenotype.

### Proof of principle: SOX9 in the developing testis

To test whether MO treatment can influence phenotype when the protein of interest is already abundant at the time of treatment, we performed MO knockdown of SOX9 (SRY (sex determining region Y)-box 9) at 11.5 dpc. SOX9 expression stimulates the male pathway by promoting Sertoli cell differentiation [24]. In *Sox9* ^-/-^ XY embryos, gonadal sex reversal occurs as SOX9 is both necessary and sufficient for male sex determination [25,26]. However, in heterozygous *Sox9*-mutant XY embryos, Sertoli cells are able to differentiate and the SOX9 downstream target anti-Müllerian hormone (AMH) is still produced [27,28]. Since SOX9 protein is already abundant in the XY genital ridge at 11.5 dpc, the time of MO treatment, we asked whether MO treatment might result in no effect, full gonadal sex reversal, or an intermediate phenotype.

We found that SOX9 protein abundance was significantly decreased in the Sox9MO treated gonads, as assessed by Western blot (Fig. 2C, for blots see S3 Fig.) and immunofluorescence (Part H in S2 Fig.), although the expression of *Sox9* transcript was unchanged (Part A in S2 Fig.). Moreover, the expression of *bona fide* direct SOX9 target genes *Amh* [29] and *Ptgds* (*prostaglandin D2 synthase*; [30]) were significantly reduced (Fig. 2D, Part B in S2 Fig.) in Sox9MO treated XY gonads compared to XY controls, and AMH protein expression levels were also reduced compared to the control XY gonad (Fig. 2E).

In Sox9MO-treated gonads, residual SOX9 and therefore AMH expression was sufficient to initiate Müllerian duct regression by 13.5 dpc (Part J in S2 Fig.). Consistent with this finding, we showed that SOX9 levels were not sufficiently suppressed as to allow upregulation of the female somatic pathway; FOXL2-positive cells were not observed (Part H in S2 Fig.) and expression of *Fst* (*follistatin*), a female somatic marker, was not upregulated in XY Sox9MO samples compared to XY cMO samples as assessed by qRT-PCR (Part F in S2 Fig.).

The expression of another Sertoli expressed gene, *Dhh* (*desert hedgehog*; [31]) was not significantly downregulated (Part C in S2 Fig.). Accordingly, fetal Leydig cell (FLC) differentiation occurred in the knockdown of SOX9 in XY gonads, as assessed by expression of FLC markers *Cyp11a1* (*cytochrome P450*, *family 11*, *subfamily a*, *polypeptide 1*; Part E in S2 Fig.), *Nr5a1/*NR5A1 (nuclear receptor subfamily 5, group A, member 1; Part D, I in S2 Fig.) and HSD3β (hydroxy-delta-5-steroid dehydrogenase, 3 beta- and steroid delta-isomerase 1; Fig. 2E). As expected, germ cells were unaffected by Sox9MO treatment in both XX and XY gonads, as assessed by the expression of *Ddx4* (Part G in S2 Fig.) and CDH1 (cadherin 1; Part I in S2 Fig.).

In summary, treatment with Sox9MO at 11.5 dpc resulted in a phenotype similar to that of the heterozygous *Sox9* genetic knockout, with reduced target gene expression but no gonadal sex reversal. There was no effect of Sox9MO treatment on germ cells, suggesting the phenotype observed was not due to off-target or toxic effects of the MO.

### Proof of principle: SOX9 in the developing pancreas

To demonstrate the utility of MO heart injections for functional assay in other developing organs, we knocked down SOX9 in the developing pancreas. In addition to its roles in gonadogenesis, SOX9 also plays a role endocrine cell differentiation in the pancreas [32,33]. Heterozygous *Sox9*-mutant mice (most closely phenocopied by the Sox9MO effects on gonadal development described above) form fewer endocrine islets, but insulin- and glucagon-positive daughter cells still differentiate [32]. Additionally, heterozygous *Sox9*-mutant mice have decreased expression of *Pdx1* (*pancreatic and duodenal homeobox 1*; expressed in SOX9-positive multipotent progenitor cells) and *Ngn3* (*neurogenin 3*; endocrine progenitor cells) [34]. We therefore investigated whether treatment with Sox9MO at 11.5 dpc would cause a decrease in expression of *Pdx1/Ngn3* and genes associated with insulin production and/or a decrease in the number of endocrine insulin-positive cells.

We conducted our analyses at 4 days and 6 days post-treatment (the equivalent of 15.5 dpc and 17.5 dpc, respectively). By immunofluorescence we saw a decrease in SOX9 expression (Part K, L in S4 Fig.) in Sox9MO treated pancreata at 15.5 dpc. Importantly, PAX6-positive (Part I, J in S4 Fig.; Fig. 2H) and INS-positive (Fig. 2I) endocrine cells were present in Sox9MO treated pancreata, indicating that the residual SOX9 expression after MO treatment at 11.5 dpc is sufficient to allow endocrine cell types to differentiate. We observed a significant decrease in the expression of *Ins1* (*insulin 1*) and *Ins2* (*insulin 2*) in Sox9MO treated pancreata at 17.5 dpc (Fig. 2F, Part D in S4 Fig.), however the number of INS-positive (Insulin I/II) cells was unperturbed (Fig. 2I). We also investigated the expression of *Pax6* (*paired box 6*), which marks endocrine cells, and found no change in *Pax6* expression (Fig. 2G) or the number of PAX6-positive cells (Fig. 2H) in response to Sox9MO treatment in the cultured pancreata. We found by qRT-PCR that expression of putative direct SOX9 target *Pdx1* (Part C in S4 Fig.; [34]) was unaltered but *Ngn3* (Part B in S4 Fig.; [34]) expression was significantly decreased at 17.5 dpc.

SOX9 knockdown partially mimicked the heterozygous *Sox9*-mutant mouse phenotype as the effect we saw on endocrine cells was restricted to expression of *Ins1*, *Ins2* and *Ngn3*. Expression of *Sox9* was unaltered (Part A in S4 Fig.), as was expression of non-β cell sub-type markers including *Glug* (*glucagon*; α-cells, Part E in S4 Fig.), *Ghrl* (*Ghrelin*; ε-cells, Part F in S4 Fig.), *Ppy* (*Pancreatic polypeptide*; PP-cells, Part G in S4 Fig.) and *Sst* (*Somatostatin*; δ-cells, Part H in S4 Fig.) at both timepoints.

Together, these results suggest that *Ins1*, *Ins2* and *Ngn3* transcription in the pancreas was specifically suppressed by Sox9MO treatment which, therefore, partially phenocopied the heterozygous *Sox9*-mutant mice [32,34]. The specificity of these effects suggests that MO treatment did not result in off-target effects or generalised toxicity in the pancreas. Moreover, the effects of MO knockdown were detectable for at least 6 days post treatment.

### MO-mediated double knockdown of GLI transcription factors

We next investigated whether this approach could be used to knock down multiple genes simultaneously, as is commonplace in zebrafish and *Xenopus* studies. To this end we created a double knockdown of the downstream Hedgehog pathway activators GLI1 (GLI-Kruppel family member 1) and GLI2 (GLI-Kruppel family member 2). The Hedgehog signaling pathway promotes the differentiation of the steroidogenic FLC population during testis development. During this process, the ligand DHH (Desert hedgehog) is secreted by Sertoli cells. Hedgehog receptor, PTCH1 (Patched homolog 1), which is induced by Hedgehog signaling, as well as Hedgehog targets GLI1 and GLI2, are expressed by cells of the entire interstitial space that surrounds the testis cords [31,35,36]. In *Dhh*-knockout XY gonads, there are greatly reduced numbers of steroidogenic FLCs [31,36]. However, the differentiation of the FLC population is unaffected in XY gonads of either of *Gli1* or *Gli2* single-knockout embryos, suggesting that GLI factors act redundantly in the testis [35].

**Fig 3:**
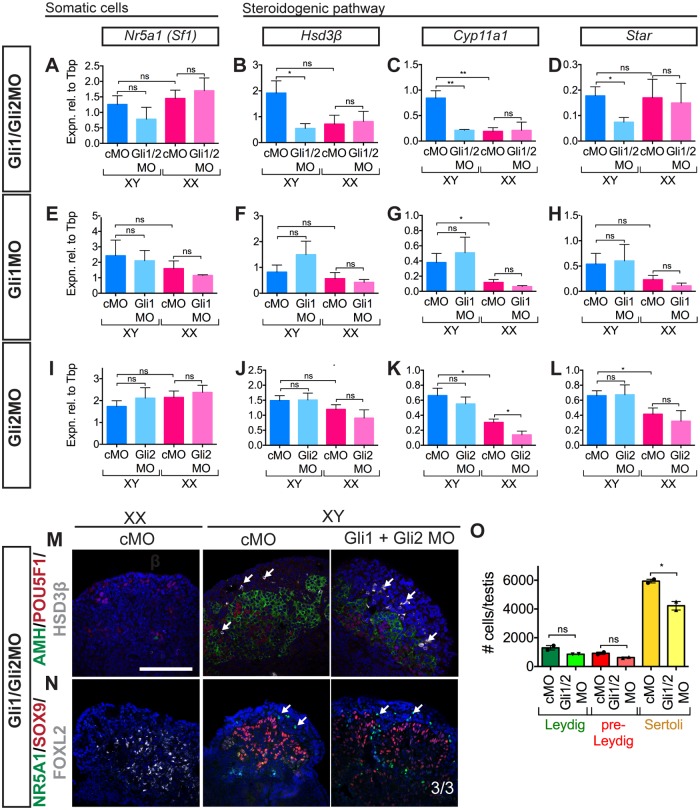
Double knockdown of *Gli1/Gli2* in XY gonads. (A–D) Knockdown of GLI1/GLI2 in the gonad: qRT-PCR showed that treatment with Gli1/Gli2MO (*n* = 6, 5, 5, 8) resulted in no significant downregulation in steroidogenic regulator *Sf1/Nr5a1* (A) but a significant downregulation in expression of steroidogenic pathway enzymes *Hsd3β* (B), *Cyp11a1* (C) and *Star* (D). No change was observed in *Nr5a1* expression in Gli1MO or Gli2MO knockdown (E, I). Similarly, there were no changes in expression of steroidogenic pathway enzymes *Hsd3β* (F, J), *Cyp11a1* (G, K) and *Star* (H, L) in Gli1MO (E–H; *n* = 6, 6, 7, 5) or Gli2MO (I–L; *n* = 8, 7, 4, 3) single knockdowns. IF showed Sertoli cells (AMH (M) and SOX9 (N)) and germ cells (POU5F1 (M)) were present in XY Gli1/Gli2MO treated gonads and no FOXL2-positive cells were observed (N). Steroidogenic *Hsd3β*-positive (M) and *Nr5a1*-positive (N) cells were still present in Gli1/Gli2MO treated XY gonads. Quantification (*n* = 2) of steroidogenic cells revealed no change in the number of HSD3β-positive Leydig cells (O; green) or SF1-positive/SOX9-negative pre-Leydig cells (O; red). There was a decrease in the number of SOX9-positve Sertoli cells in the Gli1/2MO treated XY gonads (O; yellow). Scale bars = 100 μM; cMO = control morpholino; xMO = morpholino targeting gene x. For all qRT-PCR levels are shown relative to *Tbp*, error = S.E.M. For cell quantification error = S.E.M. with individual counts plotted. * = p = 0.05, ** = p = 0.001, ns = not statistically significant.

To address this potential redundancy, we generated a double knockdown of *Gli1/Gli2* using MO heart injection at 11.5 dpc and examined the effects 48h post-injection equivalent to 13.5 dpc. As a result, we detected by qRT-PCR a decrease in expression of steroidogenic pathway genes *Nr5a1*, *Star* (*steroidogenic acute regulatory protein*), *Cyp11a1* and *Hsd3β* (Fig. 3A–D), indicating a reduction in steroidogenic cell number or capacity. The decrease in *Nr5a1* expression was consistent but not statistically significant. Notably, no change in the expression of these genes was detected in single Gli1MO (Fig. 3E–H) or Gli2MO (Fig. 3I–L) knockdowns. Thus, the attenuation of steroidogenic gene expression was specific to the Gli1/2MO double knockdown. No difference was observed in levels of the Hedgehog receptor gene *Ptch1* by qRT-PCR in the double or single knockdowns relative to controls (Part C, F, I in S5 Fig.), indicating that the extent of GLI knockdown was not sufficient to perturb expression of at least one known GLI target.

We quantified the number of steroidogenic cells to determine whether the decrease in steroidogenic gene expression was due to a decrease in cell number or to an impediment to cell maturation. There was no significant difference in the number of NR5A1-positive/SOX9-negative (immature FLC) or HSD3β-positive (FLC) cells between Gli1/2MO treated XY gonads and controls (Fig. 3M, N, O), suggesting that the observed phenotype is due to a decrease in steroidogenic capacity of the Leydig cell population. Testis cords formed properly and expression of Sertoli cell marker *Amh/*AMH (Part A, D, G in S5 Fig.) and germ cell markers *Ddx4/*POU5F1 (Fig. 2M, Part B, E, H in S5 Fig.) appeared unaffected by the Gli1/2MO treatment (Fig. 3C, D), consistent with a lack of off-target or broadly toxic effects. Our results support functional redundancy between *Gli1* and *Gli2* in FLCs, and demonstrate proof-of-principle that heart injection of MO can be used to target multiple genes simultaneously to assess possible genetic interactions.

### Addressing novel gene function: Adamts19 and Ctrb1

Finally, we characterised the knockdown of two genes to which functions have not previously been ascribed, so as to test the utility of the system for first-pass functional characterisation of novel genes. We focused first on the ovarian gene *Adamts19* (*a disintegrin-like and metallopeptidase [reprolysin type] with thrombospondin type 1 motif*, *19*), identified in a PCR-based cDNA subtraction screen, and in which polymorphisms have since been associated with premature ovarian failure (POF; [37–39]. The function of this gene remains unknown at the molecular, cellular or whole organism levels.

**Fig 4:**
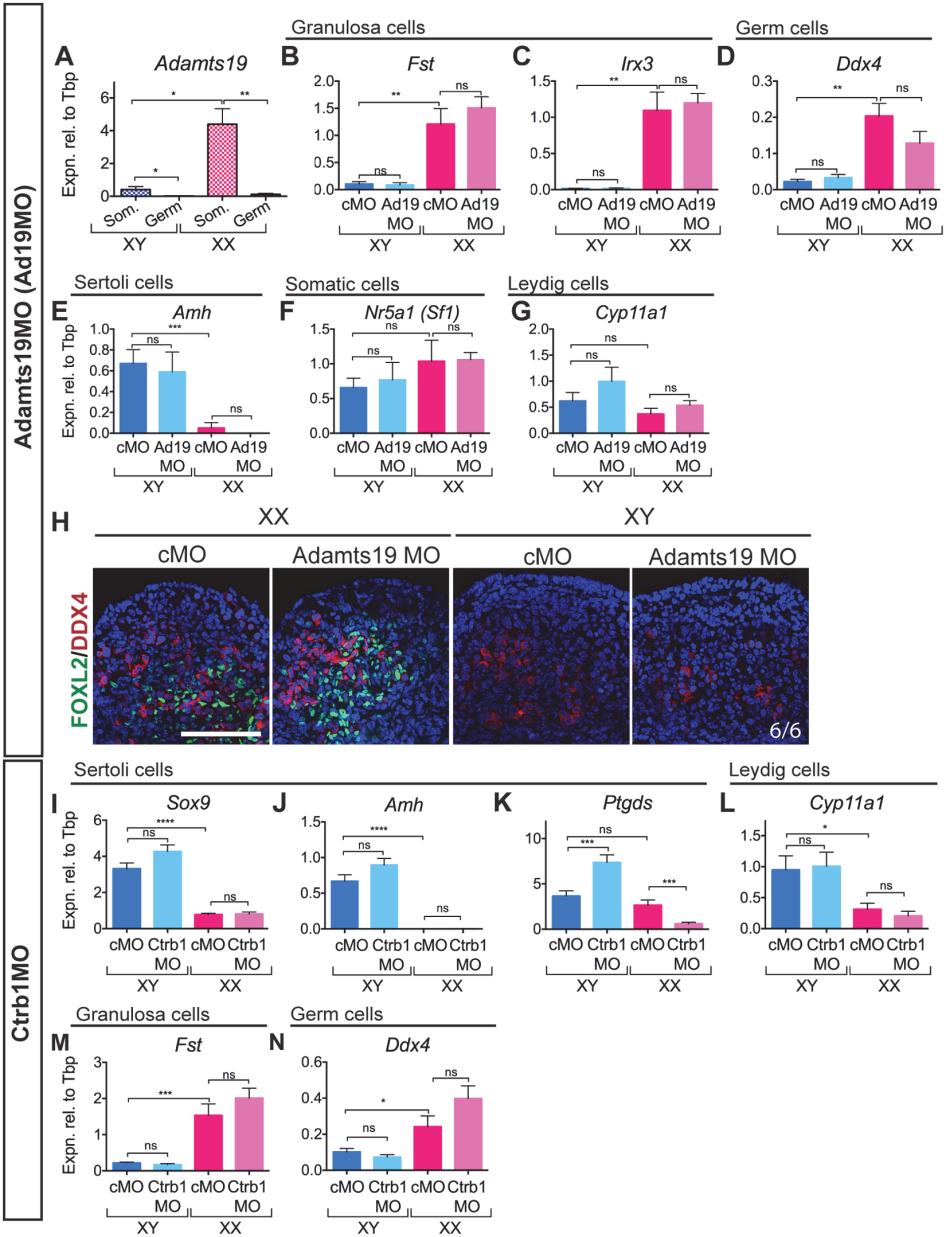
Knockdown of *Adamts19* in XX gonads and *Ctrb1* in XY gonads. (A) qRT-PCR on FACS-sorted somatic and germ cells (*n* = 3, 4, 3, 4) shows that *Adamts19* is expressed in the somatic cells of the ovary at 12.5 dpc and at much lower levels in somatic cells of the testis. Knockdown of ADAMTS19 in the XX gonad (n = 7, 8, 5, 5) showed no change in female somatic markers *Fst* (B) and *Irx3* (C) and a slight decrease in expression of germ cell marker *Ddx4* (D). Male markers, *Amh* (Sertoli cells; E), *Nr5a1* (Somatic cells; F) and *Cyp11a1* (Leydig cells; G) were unperturbed. IF showed no discernable difference in the ratios of FOXL2-positive/DDX4-positive cells in the Adamts19MO-treated XX gonad compared to the control (H). Knockdown of CTRB1 in the XY gonad (*n* = 19, 16, 14, 14) resulted in no change to male somatic markers *Sox9* (I) or *Amh* (J) but an increase in *Ptgds* (K) was observed in the Ctrb1MO-treated XY gonad. Expression of Leydig cell marker *Cyp11a1* (L), female somatic marker *Fst* (M) and germ cell marker *Ddx4* (N) was unchanged. Germ = germ cells, Som. = somatic cells. Scale bars = 100 μM; cMO = control morpholino; xMO = morpholino targeting gene x. For all qRT-PCR: levels are shown relative to *Tbp*, error = S.E.M., * = p = 0.05, ** = p = 0.001, *** = p = 0.0001, **** = p = 0.00001, ns = not statistically significant.

We performed qRT-PCR on FACS-sorted somatic cells at 12.5 dpc and confirmed that *Adamts19* was expressed in FOXL2-positive somatic cells, and not in the XX germ cells (Fig. 4A). MO knockdown of *Adamts19* resulted in no change in XX granulosa somatic markers *Fst* or *Irx3* (*Iroquois related homeobox 3*; Fig. 4B, C) and slight but not statistically significant decrease in expression of the germ cell marker *Ddx4* (Fig. 4D). However, there were no observed gross changes in the ratio of the number of FOXL2-positive (somatic) to MVH-positive (germ) cells by immunofluorescence (Fig. 4H). qRT-PCR expression of male markers *Amh* (Fig. 4E) and *Cyp11a1* (Fig. 4G) and somatic marker *Nr5a1* (Fig. 4F) were unperturbed by Adamts19MO treatment indicating there were no broad off-target effects of MO treatment. These results do not indicate a clear role for *Adamts19* in the developing ovary. Importantly, these data illustrate that treatment with a MO does not always perturb gonadogenesis, pointing to a lack of generalized non-specific artefacts.

We also examined the Sertoli-expressed gene *Ctrb1* (*chymotrypsinogen B1*), which has been implicated in gonadal development. In a screen of XX *Wnt4*-knockout (*wingless-related MMTV integration site 4*) mice, which exhibit partial sex reversal, expression of *Ctrb1* was increased, suggesting an association with the testis development pathway [40]. Differential expression data sets indicate that *Ctrb1* is testis-specific from 12.5 dpc and that it is expressed in the Sertoli cell lineage [41].

Knockdown of *Ctrb1* resulted in no change to Sertoli cell markers *Sox9* and *Amh*, but a statistically significant increase in the expression of *Ptgds* in the testis by qRT-PCR (Fig. 4I, J, K). In the XX Ctrb1MO treated gonad, *Ptgds* expression was decreased compared to the XX control. No changes were observed in the expression of the steroidogenic gene *Cyp11a1*, granulosa cell marker *Fst* or germ cell marker *Ddx4* (Fig. 4L, M, N) in the XY Ctrb1MO treated gonad, suggesting that the other testis cell lineages are unperturbed. The increase in *Ptgds* expression resulting from knockdown of *Ctrb1* implicates *Ctrb1* in processes downstream of SOX9, such as *Ptgds* regulation and as such provides a basis for the instigation of further genetic studies.

## Discussion

We describe here a novel first-pass screening method that can provide insights into the function of candidate organogenesis genes, singly or in combination, either to assist with the design of in-depth genetic and biochemical investigations, or to prioritize lists of candidate genes for these investigations. By injection of MOs into the heart of mouse embryos, we exploited the embryonic vasculature to deliver the MO to the target tissues, which were then explanted, cultured and analysed. Using this technique we partially reproduced known gene knockout phenotypes in the fetal gonads and pancreas, created a novel double knockdown of GLI1 and GLI2, and screened *Adamts19* and *Ctrb1* for potential function in early gonadal development. These studies reveal the utility of this method to obtain insights into gene function during organogenesis rapidly and relatively simply.

The method described here provides a significant improvement on previous injection- and electroporation-based delivery strategies, which suffered from limited delivery area and/or uptake, tendency for tissue damage and lack of reproducibility. Published methods of gain-of-function (cDNA) or loss-of-function (shRNA) construct delivery by magnetofection, nucleofection or liposome-mediated methods in cultured gonads have shown delivery of the effector construct to 2–20% of cells in the target tissue [2–4,42]. In contrast, we visualized delivery of fluorescent MO throughout the tissue of interest, saw consistent knockdown of downstream target genes throughout the cultured organ, and showed in the XY gonad that the MO could target genes in multiple cell lineages. Secondly, injection of the MO into the heart avoids compromising the integrity of the target tissue by direct contact with needles and/or electrodes. Finally, relying on systemic delivery rather than direct injection of the effector construct avoids experimental error and instead produced consistent gene knockdown for the target gene in multiple experiments performed over a two-year period.

Encouragingly, in our proof-of-principle and double-knockdown experiments, it was the capacity of a cell population to express downstream target genes and proteins, rather than the number of expressing cells, that was altered by MO treatment. The knockdown of the target protein was incomplete in all cases; this allowed differentiation of the target cells but their functionality was reduced. For example, FLCs still differentiated in normal numbers in the *Gli1/Gli2* MO treated XY gonads, but they did not produced steroid enzymes at the same capacity as the controls. This indicates that the processes controlled by GLI factors were being perturbed by MO treatment, similarly to the Sox9MO treated XY gonads and pancreata. Importantly, the subtle outcomes of MO treatment were highly reproducible, as shown by our qRT-PCR analyses, suggesting that the information generated provides a robust basis on which to base mechanistic hypotheses and further experiments.

In addition to partially reproducing several established null mouse models, using MO injection we strengthened the case for creating a complex genetic conditional double knockout of GLI1 and GLI2 in FLCs [35]. Our findings suggest that there is functional redundancy between GLI1 and GLI2 in the developing testis and that further genetic analysis is likely to be fruitful.

With any experiments involving MOs, careful attention to controls is required [43]. By careful examination of untargeted cell populations in the organ of interest, we were able to identify and exclude off-target effects and toxicity. Nonetheless, concerns have been raised regarding the difference between MO knockdown phenotypes and other functional analysis methods [44]. This difference is at least partly explained by the fact that MO knockdown only partially reduces overall activity of the target protein; certainly, in our Sox9MO experiments, the phenotypes obtained more closely resembled heterozygous than homozygous knockouts. All things considered, it is clear that genetic targeting by homologous recombination or CRISPR/Cas9 approaches will remain the gold standard for functional analysis. Therefore, we suggest that, once a likely effect is revealed by MO studies, it would be more useful to advance to definitive functional experiments, rather than to devote additional resources to definitively excluding off-target effects (for example by assaying multiple MOs for each gene of interest).

## Supporting Information

S1 Video Demonstration of heart injection of constructs in 11.5 dpc embryo. This video demonstrates the injection of a construct (marked by blue dye) into the left ventricle of the beating embryo heart at 11.5 dpc. After several heartbeats the dye can be seen in more distal parts of the embryo and finally in the head vein indicating successful injection. After injection the embryo is incubated with the heart still beating for 30 min before dissection for organ culture. For more detailed information see Fig. 1 and Materials and Methods.
(MP4)

S1 Fig Knockdown of STRA8 does not affect general markers of gonadal or germ cell development. Gene expression profiled by qRT-PCR in cMO-treated (XX and XY) versus Stra8MO- treated XX gonads (*n* = 4, 4, 10, 14) showed that target gene *Stra8* (A) and female marker gene *FoxL2* (B) were unchanged. Similarly, meiosis marker genes *Dmc1* (DMC1 dosage suppressor of mck1 homolog, meiosis-specific homologous recombination; C) *Scp3*, (D) and *Rec8* (REC8 meiotic recombination protein; E) and *g*erm cell marker genes *Ddx4* (F), *Pou5f1* (G) and *Cdh1* (H) were unperturbed. IF for CDH1 and FOXL2 indicated that germ cells and somatic cells are present in Stra8MO-treated XX gonads (I; *n* = 3). Scale bars = 100 μM; cMO = control morpholino; xMO = morpholino targeting gene x. For all qRT-PCR levels are shown relative to *Tbp*, error = S.E.M., * = p = 0.05, ** = p = 0.001, ns = not statistically significant.
(TIF)

S2 Fig Knockdown of SOX9 using Sox9MO in gonad is specific to Sertoli cells but does not cause sex reversal. qRT-PCR showed that knockdown of SOX9 in the gonad (A, B: *n* = 8, 15, 11, 4; C–G: *n* = 5, 9, 6, 4) had no apparent effect on target gene *Sox9* (A), however, downregulation of expression of SOX9 target gene *Ptgds* (B) was observed. Levels of Sertoli gene *Dhh* (C), somatic gene *Nr5a1* (D), FLC marker *Cyp11a1* (E) were unperturbed in Sox9MO-treated gonads. While expressed at very low levels in XY gonads, ovarian marker *Fst* (F) was significantly decreased in XY Sox9MO-treated gonads. Expression of germ cell marker *Ddx4* (G) was unperturbed. IF of XY Sox9MO treated gonads showed a decrease in SOX9 expression with no evidence of sex reversal (FOXL2-positive cells) (H; *n* = 5). Germ cells (CDH1) and FLCs (NR5A1) could be observed in XY Sox9MO treated gonads by IF (I). Whole-mount IF of gonad mesonephroi staining (J; *n* = 3): PAX2 (paired box 2), marks the Müllerian duct (MD), Wolffian duct (WD) and mesonephric tubules, and CDH1, marks the Wolffian duct and mesonephric tubules. The Müllerian duct is not retained in XY Sox9MO-treated mesonephroi indicating that the low level of AMH present can regress the duct as normal. Scale bars = 100 μM; cMO = control morpholino; xMO = morpholino targeting gene x. For all qRT-PCR: levels are shown relative to *Tbp*, error = S.E.M., * = p = 0.05, ** = p = 0.001, *** = p = 0.0001, **** = p = 0.00001, ns = not statistically significant.
(TIF)

S3 Fig Raw Western blots showing knockdown of SOX9 in the Sox9MO treated XY gonad. (AF) Western blot for SOX9 (relative to α-TUBULIN or β-ACTIN) showed a downregulation of SOX9 upon Sox9MO treatment in XY gonads (*n* = 3) quantified in Fig. 2C. For Western blots SOX9 levels (B, D, F) were normalised to α-TUBULIN or β-ACTIN loading controls for each blot (A, C, E) and Sox9MO-treated XY gonads measured relative to cMO treated XY gonads with expression for each blot set to 1. 13.5 dpc XY gonads were used as a positive control and 13.5 dpc XX gonads were used as a negative control for SOX9 antibody specificity. cMO = control morpholino; xMO = morpholino targeting gene x.
(TIF)

S4 Fig Knockdown controls for in Sox9MO treated pancreata. qRT-PCR (*n* = 5, 5, 5, 5) showed that *Sox9* (A) expression was unperturbed by Sox9MO-treatment. Expression of *Ngn3* (B; marker of multipotent progenitor cells (MPCs)) was significantly decreased at 17.5 dpc. Expression of *Pdx1* (C; marker of endocrine progenitor cells (EPCs)) was unaltered, but *Ins2* (D) expression was significantly decreased in the Sox9MO-treated pancreata at 17.5 dpc. Expression of non-β-cell sub-type markers: α-cells *Glug* (E), ε-cells *Ghrl* (F), PP-cells *Ppy* (G) and δ-cells *Sst* (H) were all unaltered by treatment with Sox9MO. IF at 15.5 dpc showed that as in the cMO-treated pancreata (I), PAX6-positive cells (indicated by white arrows) differentiate when treated with Sox9MO (J), however, SOX9 expression (K, L; indicated by white arrows) is diminished when treated with Sox9MO. cMO = control morpholino; xMO = morpholino targeting gene x. For all qRT-PCR: levels are shown relative to *Tbp*, error = S.E.M., * = p = 0.05, ** = p = 0.001, ns = not statistically significant.
(TIF)

S5 Fig Gli1/2MO treatment has no effect of Sertoli or germ cells. (A-C) Knockdown of GLI1/GLI2 in the gonad (*n* = 6, 5, 5, 8): qRT-PCR for Sertoli cells marked by *Amh* (A), germ cells marked by *Ddx4* (B) and hedgehog receptor *Ptch1* (C) showed no change after Gli1/Gli2MO treatment. The same trend was observed in the Gli1MO knockdown (*n* = 6, 6, 7, 5; *Amh* (D); *Mvh* (E); *Ptch1* (F)) and the Gli2MO knockdown (*n* = 8, 7, 4, 3; *Amh* (G); *Mvh* (H); *Ptch1* (I)). cMO = control morpholino; xMO = morpholino targeting gene x. For all qRT-PCR levels are shown relative to *Tbp*, error = S.E.M., * = p = 0.05, ns = not statistically significant.
(TIF)

S1 Table Splice site MO sequences targeting exon/intron boundaries of target genes. Morpholino sequences for targets described in manuscript.
(DOCX)

S2 Table Taqman gene expression sets for qRT-PCR. TaqMan Gene Expression Assay catalogue numbers described in manuscript.
(DOCX)

S3 Table Primary Antibodies for Immunofluorescence and Western Blot. Dilutions and catalogue numbers for primary antibodies described in manuscript.
(DOCX)

S4 Table Secondary Antibodies for Immunofluorescence and Western Blot. Dilutions and catalogue numbers for secondary antibodies described in manuscript.
(DOCX)
