# Supplementary figures and images for: Rapid Screening of Gene Function by Systemic Delivery of Morpholino Oligonucleotides to Live Mouse Embryos
Source: PLoS One. 2015 Jan 28;10(1):e0114932. doi: 10.1371/journal.pone.0114932 (PMC4309589; doi:10.1371/journal.pone.0114932)

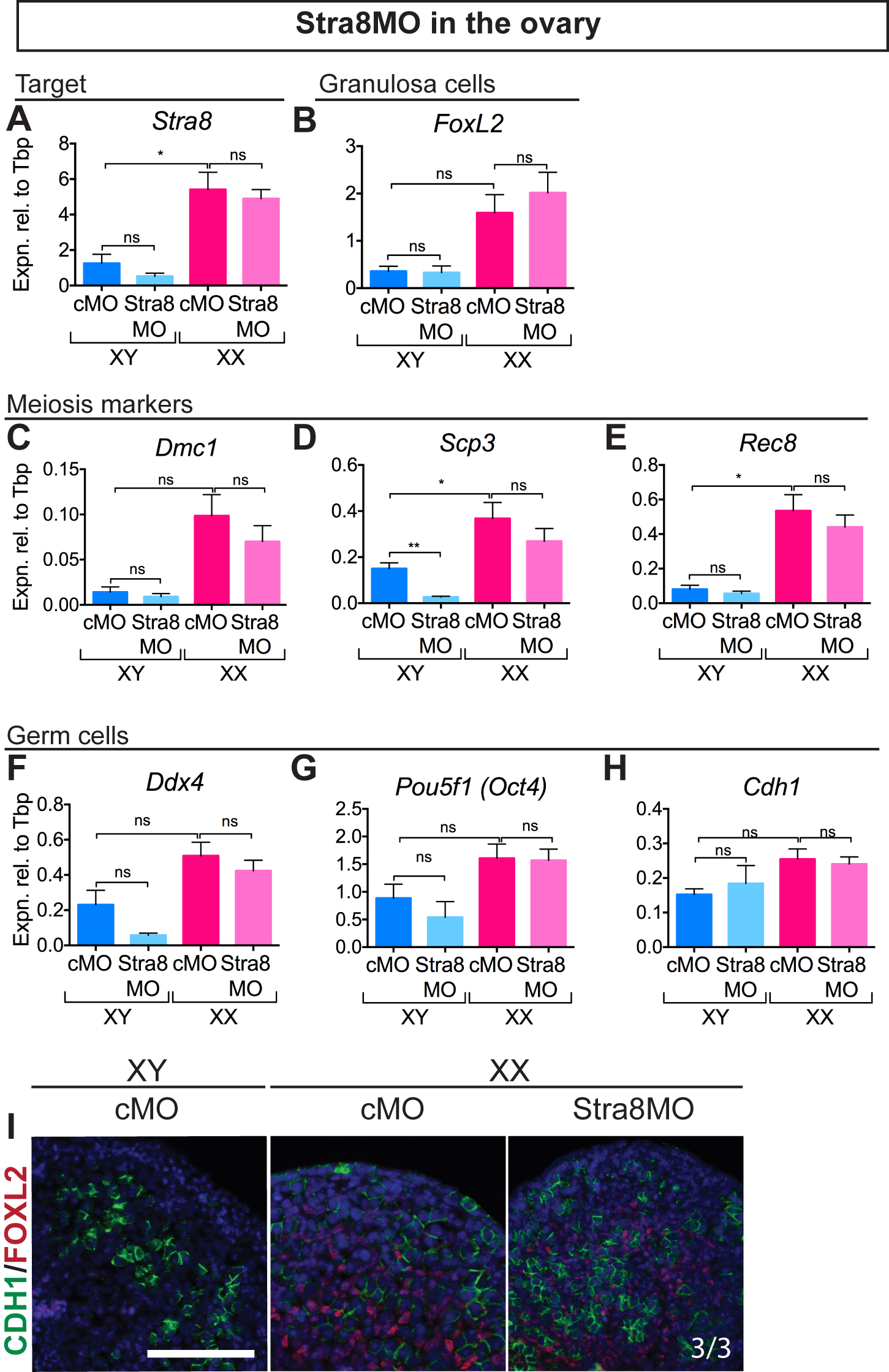

Supplement: S1 Fig — Gene expression profiled by qRT-PCR in cMO-treated (XX and XY) versus Stra8MO- treated XX gonads (n = 4, 4, 10, 14) showed that target gene Stra8 (A) and female marker gene FoxL2 (B) were unchanged. Similarly, meiosis marker genes Dmc1 (DMC1 dosage suppressor of mck1 homolog, meiosis-specific homologous recombination; C) Scp3, (D) and Rec8 (REC8 meiotic recombination protein; E) and germ cell marker genes Ddx4 (F), Pou5f1 (G) and Cdh1 (H) were unperturbed. IF for CDH1 and FOXL2 indicated that germ cells and somatic cells are present in Stra8MO-treated XX gonads (I; n = 3). Scale bars = 100 μM; cMO = control morpholino; xMO = morpholino targeting gene x. For all qRT-PCR levels are shown relative to Tbp, error = S.E.M., * = p = 0.05, ** = p = 0.001, ns = not statistically significant. (TIF) [file pone.0114932.s002.tif]

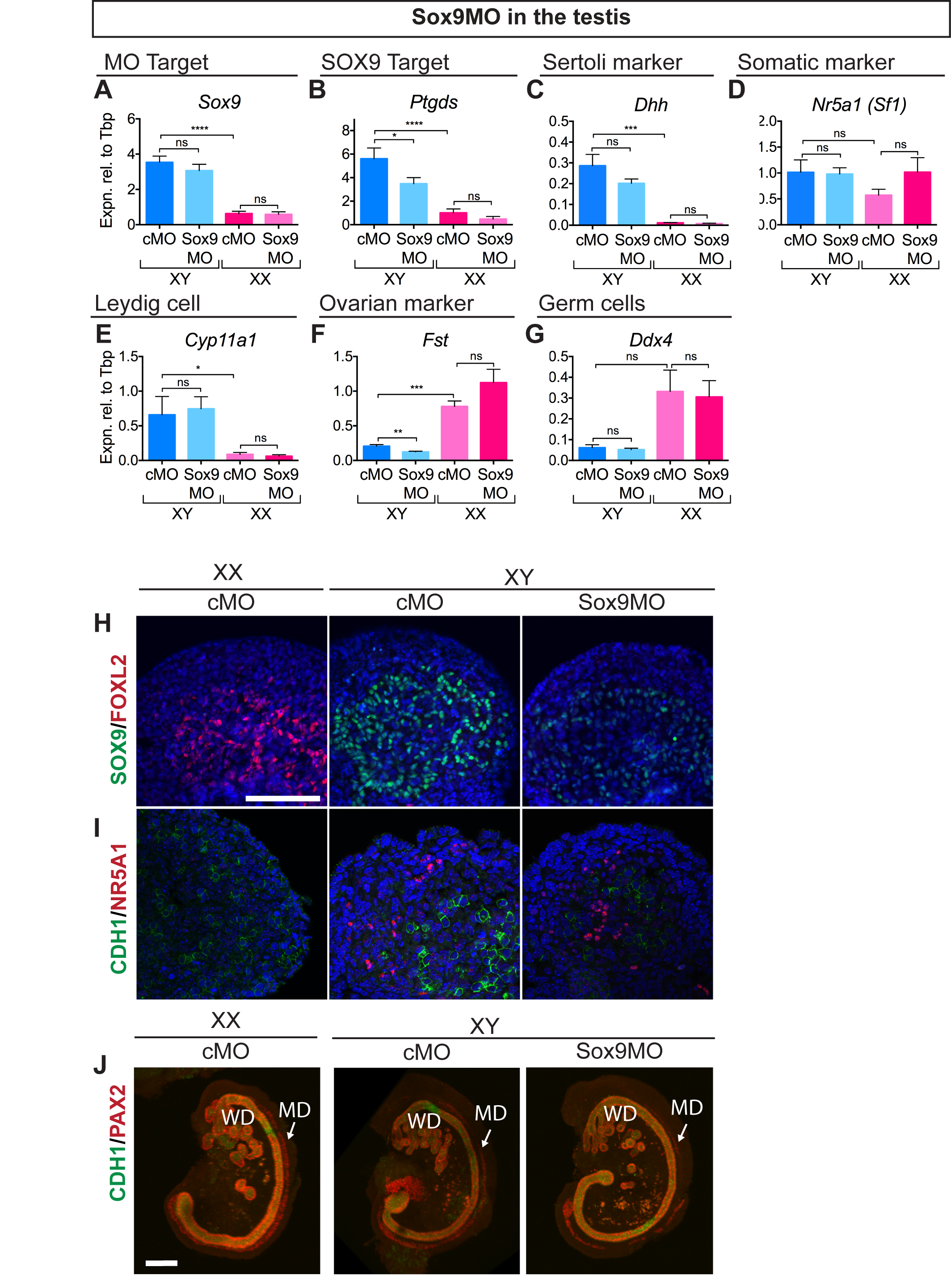

Supplement: S2 Fig — qRT-PCR showed that knockdown of SOX9 in the gonad (A, B: n = 8, 15, 11, 4; C–G: n = 5, 9, 6, 4) had no apparent effect on target gene Sox9 (A), however, downregulation of expression of SOX9 target gene Ptgds (B) was observed. Levels of Sertoli gene Dhh (C), somatic gene Nr5a1 (D), FLC marker Cyp11a1 (E) were unperturbed in Sox9MO-treated gonads. While expressed at very low levels in XY gonads, ovarian marker Fst (F) was significantly decreased in XY Sox9MO-treated gonads. Expression of germ cell marker Ddx4 (G) was unperturbed. IF of XY Sox9MO treated gonads showed a decrease in SOX9 expression with no evidence of sex reversal (FOXL2-positive cells) (H; n = 5). Germ cells (CDH1) and FLCs (NR5A1) could be observed in XY Sox9MO treated gonads by IF (I). Whole-mount IF of gonad mesonephroi staining (J; n = 3): PAX2 (paired box 2), marks the Müllerian duct (MD), Wolffian duct (WD) and mesonephric tubules, and CDH1, marks the Wolffian duct and mesonephric tubules. The Müllerian duct is not retained in XY Sox9MO-treated mesonephroi indicating that the low level of AMH present can regress the duct as normal. Scale bars = 100 μM; cMO = control morpholino; xMO = morpholino targeting gene x. For all qRT-PCR: levels are shown relative to Tbp, error = S.E.M., * = p = 0.05, ** = p = 0.001, *** = p = 0.0001, **** = p = 0.00001, ns = not statistically significant. (TIF) [file pone.0114932.s003.tif]

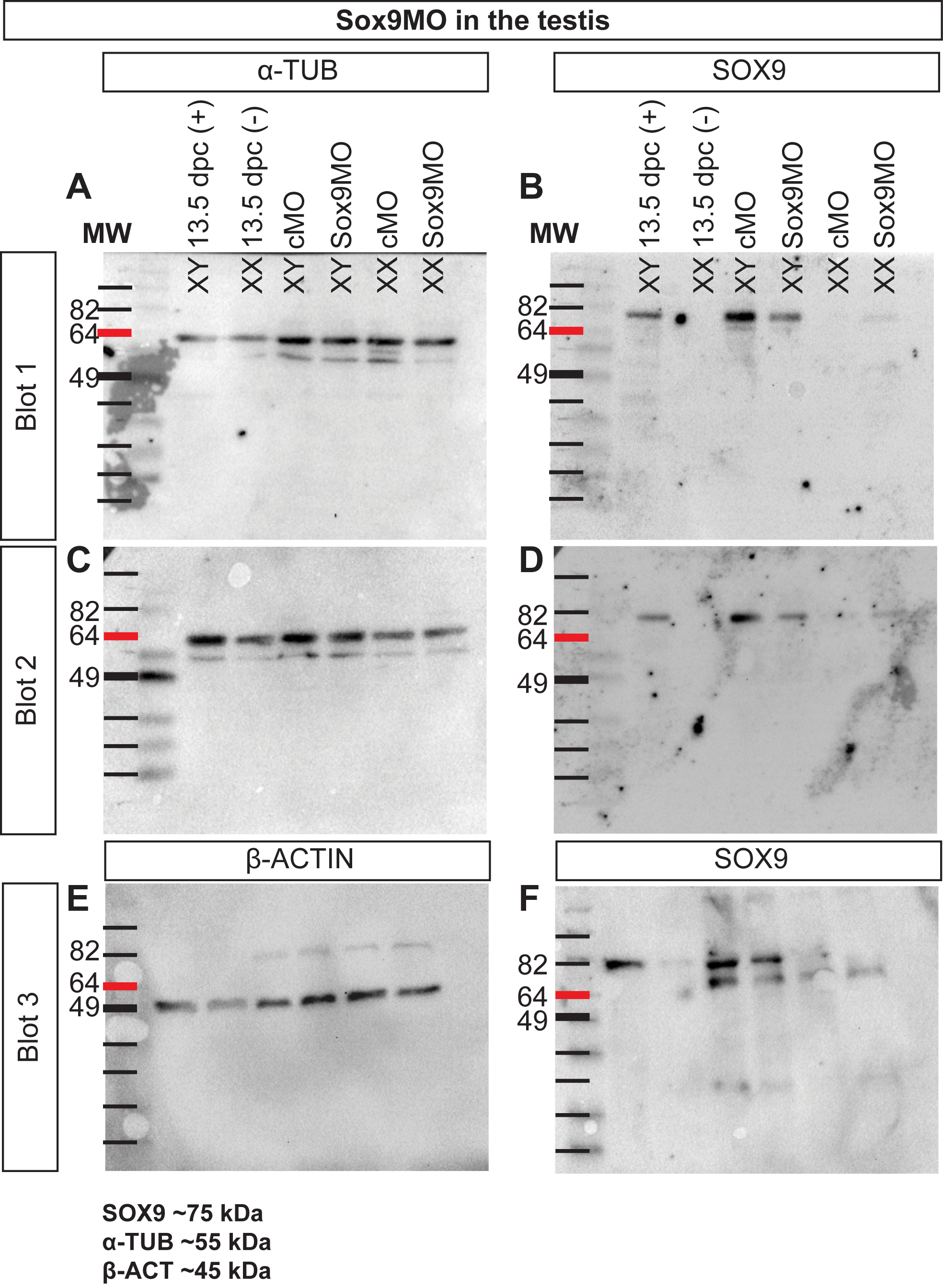

Supplement: S3 Fig — (AF) Western blot for SOX9 (relative to α-TUBULIN or β-ACTIN) showed a downregulation of SOX9 upon Sox9MO treatment in XY gonads (n = 3) quantified in Fig. 2C. For Western blots SOX9 levels (B, D, F) were normalised to α-TUBULIN or β-ACTIN loading controls for each blot (A, C, E) and Sox9MO-treated XY gonads measured relative to cMO treated XY gonads with expression for each blot set to 1. 13.5 dpc XY gonads were used as a positive control and 13.5 dpc XX gonads were used as a negative control for SOX9 antibody specificity. cMO = control morpholino; xMO = morpholino targeting gene x. (TIF) [file pone.0114932.s004.tif]

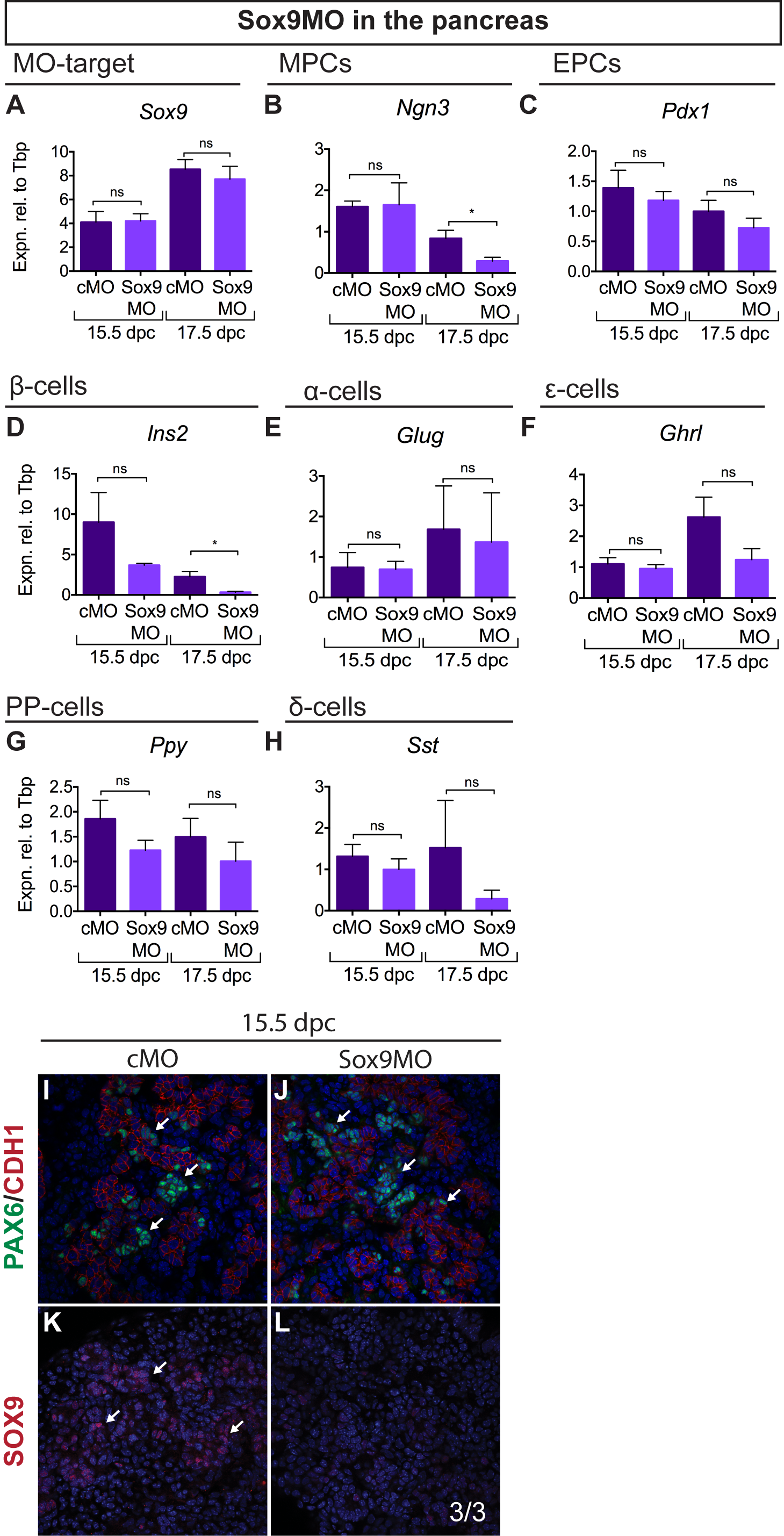

Supplement: S4 Fig — qRT-PCR (n = 5, 5, 5, 5) showed that Sox9 (A) expression was unperturbed by Sox9MO-treatment. Expression of Ngn3 (B; marker of multipotent progenitor cells (MPCs)) was significantly decreased at 17.5 dpc. Expression of Pdx1 (C; marker of endocrine progenitor cells (EPCs)) was unaltered, but Ins2 (D) expression was significantly decreased in the Sox9MO-treated pancreata at 17.5 dpc. Expression of non-β-cell sub-type markers: α-cells Glug (E), ε-cells Ghrl (F), PP-cells Ppy (G) and δ-cells Sst (H) were all unaltered by treatment with Sox9MO. IF at 15.5 dpc showed that as in the cMO-treated pancreata (I), PAX6-positive cells (indicated by white arrows) differentiate when treated with Sox9MO (J), however, SOX9 expression (K, L; indicated by white arrows) is diminished when treated with Sox9MO. cMO = control morpholino; xMO = morpholino targeting gene x. For all qRT-PCR: levels are shown relative to Tbp, error = S.E.M., * = p = 0.05, ** = p = 0.001, ns = not statistically significant. (TIF) [file pone.0114932.s005.tif]

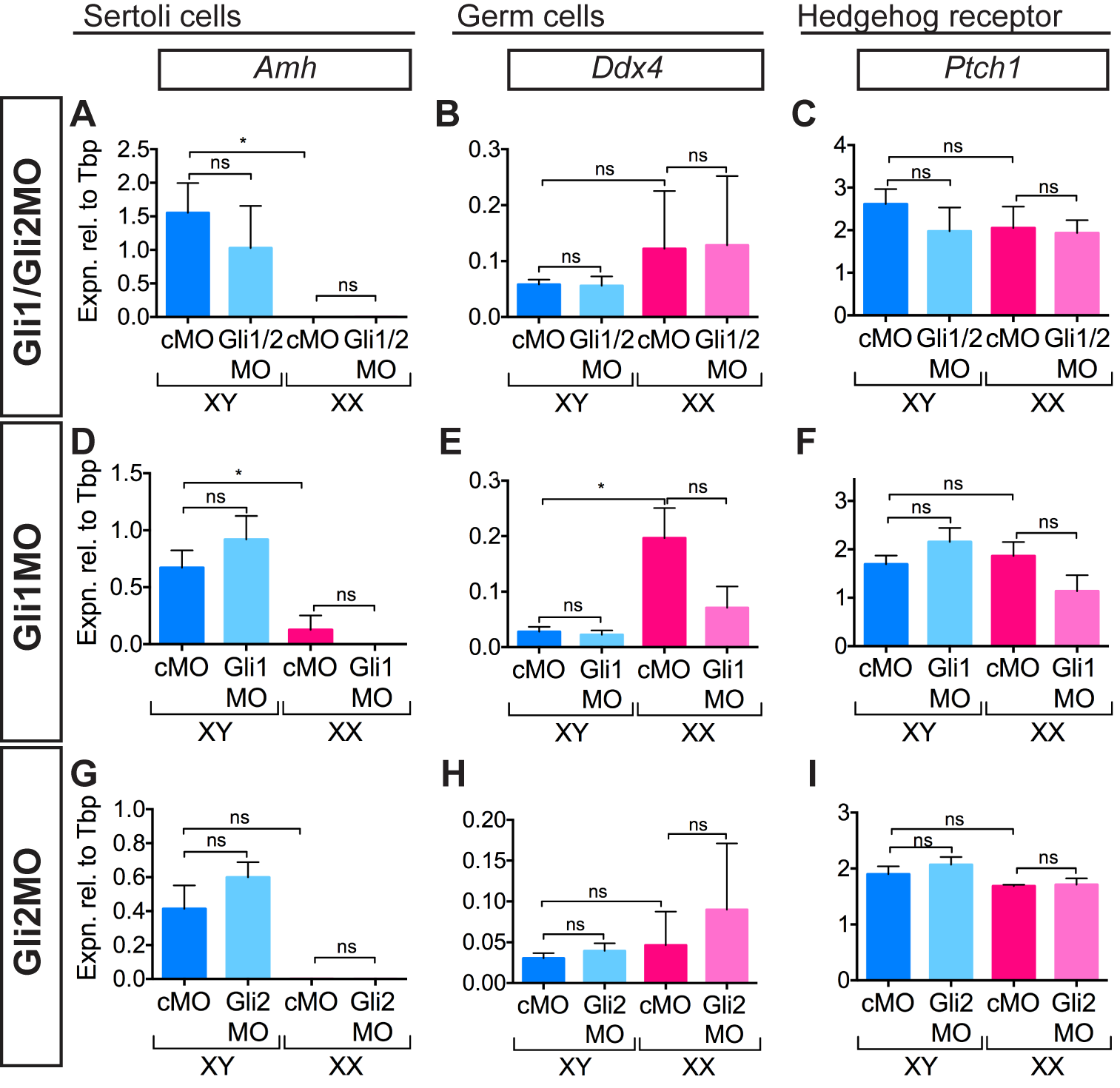

Supplement: S5 Fig — (A-C) Knockdown of GLI1/GLI2 in the gonad (n = 6, 5, 5, 8): qRT-PCR for Sertoli cells marked by Amh (A), germ cells marked by Ddx4 (B) and hedgehog receptor Ptch1 (C) showed no change after Gli1/Gli2MO treatment. The same trend was observed in the Gli1MO knockdown (n = 6, 6, 7, 5; Amh (D); Mvh (E); Ptch1 (F)) and the Gli2MO knockdown (n = 8, 7, 4, 3; Amh (G); Mvh (H); Ptch1 (I)). cMO = control morpholino; xMO = morpholino targeting gene x. For all qRT-PCR levels are shown relative to Tbp, error = S.E.M., * = p = 0.05, ns = not statistically significant. (TIF) [file pone.0114932.s006.tif]
