# Supplementary material for: Rapid Screening of Gene Function by Systemic Delivery of Morpholino Oligonucleotides to Live Mouse Embryos
Source: PLoS One. 2015 Jan 28;10(1):e0114932. doi: 10.1371/journal.pone.0114932 (PMC4309589; doi:10.1371/journal.pone.0114932)
Supplement: S1 Table — Morpholino sequences for targets described in manuscript. (DOCX) [file pone.0114932.s007.docx]

**Table S1: Splice site MO sequences targeting exon/intron boundaries of target genes**

| **Gene target** | **Antisense Sequence** |
| --- | --- |
| Adamts19 | 5'AGCTGTGGATGCTTACCAGGCCACC |
| Ctrb1 | 5'CAACGTAGCCTGGGACTCACTTGAC |
| Gli1 | 5'GGGATTGCCCCAGTGCTCACCTTCA |
| Gli2 | 5'CCACTGTCACAGGAGGCAAGAGAAA |
| Sox9 | 5'GACCACTCGCGCCTTGCTCACCAGA |
| Stra8 | 5'ACTATCCCCAAGTCCCTGTACCTTT |
| Standard Control | 5'CCTCTTACCTCAGTTACAATTTATA |
