# Supplementary material for: Rapid Screening of Gene Function by Systemic Delivery of Morpholino Oligonucleotides to Live Mouse Embryos
Source: PLoS One. 2015 Jan 28;10(1):e0114932. doi: 10.1371/journal.pone.0114932 (PMC4309589; doi:10.1371/journal.pone.0114932)
Supplement: S2 Table — TaqMan Gene Expression Assay catalogue numbers described in manuscript. (DOCX) [file pone.0114932.s008.docx]

**Table S2: Taqman gene expression sets for qRT-PCR**

| **Gene** | **Catalogue Code** | **Gene** | **Catalogue Code** |
| --- | --- | --- | --- |
| Adamts19 | Mm00558559_m1 | Notch2 | Mm00803077_m1 |
| Amh | Mm03023963_m1 | Nr5a1 (Sf1) | Mm00446826_m1 |
| Cdh1 (Ecad) | Mm01247357_m1 | Pax6 | Mm00443081_m1 |
| Cyp11a1 | Mm00490735_m1 | Pdx1 | Mm00435565_m1 |
| Ddx4 (Mvh) | Mm00802445_m1 | Pou5f1 | Mm00658129_gH |
| Dhh | Mm01310203_m1 | Ppy | Mm00435889_m1 |
| Dmc1 | Mm00494485_m1 | Ptc1 | Mm00436026_m1 |
| FoxL2 | Mm00843544_s1 | Ptgds | Mm01330613_m1 |
| Fst | Mm00514982_m1 | Rec8 | Mm00490939_m1 |
| Ghrl | Mm00445450_m1 | Scp3 | Mm00488519_m1 |
| Glug | Mm00801712_m1 | Sox9 | Mm00448840_m1 |
| Hsd3β | Mm01261921_mH | Sst | Mm00436671_m1 |
| Ins1 | Mm01950294_s1 | Star | Mm00441558_m1 |
| Ins2 | Mm00731595_gH | Stra8 | Mm00486473_m1 |
| Irx3 | Mm00500463_m1 | Tbp | Mm00446973_m1 |
|  |  |  |  |
