# Supplementary material for: Rapid Screening of Gene Function by Systemic Delivery of Morpholino Oligonucleotides to Live Mouse Embryos
Source: PLoS One. 2015 Jan 28;10(1):e0114932. doi: 10.1371/journal.pone.0114932 (PMC4309589; doi:10.1371/journal.pone.0114932)
Supplement: S3 Table — Dilutions and catalogue numbers for primary antibodies described in manuscript. (DOCX) [file pone.0114932.s009.docx]

**Table S3: Primary Antibodies for Immunofluorescence and Western Blot**

| **Primary Antibody** | **Company** | **Catalogue Code** | **Species** | **Dilution** |
| --- | --- | --- | --- | --- |
| AMH | Santa Cruz Biotechnology | sc-6886 (MIS C-20) | goat | 1:200 |
| A-TUB | Sigma | T5168 | mouse | 1:5000 (WB) |
| B-ACTIN | Thermo Scientific | PIEMA5-15739 | mouse | 1:5000 (WB) |
| CDH1 (ECAD) | BD Pharmingen 610182 | 610182 | mouse | 1:200 |
| DDX4 (MVH) | mAB Abcam | ab27591 | mouse | 1:500 |
| FOXL2 | (Polanco et al., 2010) | | rabbit | 1:800 |
| gH2AX | Millipore | 05-636 | mouse | 1:200 |
| HSD3B | Transgenic Inc | KAL-KO607 | rabbit | 1:600 |
| INS | Sigma Aldrich | 12018 | mouse | 1:200 |
| NR5A1 | Transgenic Inc | KAL-KO610 | rat | 1:600 |
| PAX2 | Invitrogen | 71-6000 | rabbit | 1:200 |
| PAX6 | Covance Research Products Inc | PRB278P | rabbit | 1:200 |
| POU5F1 | Santa Cruz Biotechnology | sc-5279 | mouse | 1:200 |
| SCP3 | Abcam ab15093 | ab15093 | rabbit | 1:200 |
| SOX9 | Abnova | H00006662-M01 | mouse | 1:200 (IF)/ 1:1000 (WB) |
| STRA8 | Abcam | ab49405 | rabbit | 1:200 |
