# Supplementary material for: Rapid Screening of Gene Function by Systemic Delivery of Morpholino Oligonucleotides to Live Mouse Embryos
Source: PLoS One. 2015 Jan 28;10(1):e0114932. doi: 10.1371/journal.pone.0114932 (PMC4309589; doi:10.1371/journal.pone.0114932)
Supplement: S4 Table — Dilutions and catalogue numbers for secondary antibodies described in manuscript. (DOCX) [file pone.0114932.s010.docx]

**Table S4: Secondary Antibodies for Immunofluorescence and Western Blot**

| **Conjugate** | **Invitrogen Catalogue Code (all used at 1:200)** | |
| --- | --- | --- |
| anti-goat 488 | A11055 | |
| anti-mouse 488 | A11001/A11017 | |
| anti-mouse 594 | A11032/A11005 | |
| anti-mouse 647 | A31571 | |
| anti-mouse HRP | Sigma: A8924 (1:2000 for WB) | |
| anti-rabbit 488 | A11034 | |
| anti-rabbit 568 | A10042 | |
| anti-rabbit 594 | A11037 | |
| anti-rabbit 647 | A31573 | |
| anti-rat 488 | A11006 | |
| anti-rat 594 | A11007 | |
|  | | |
| **Probe** | **Company** | **Dilution** |
| DAPI | Molecular Probes | 2 ng/μl in PBS at 1:5000 |
